# Assessment of Static Plantar Pressure, Stabilometry, Vitamin D and Bone Mineral Density in Female Adolescents with Moderate Idiopathic Scoliosis

**DOI:** 10.3390/ijerph17062167

**Published:** 2020-03-24

**Authors:** Liliana Cațan, Simona Cerbu, Elena Amaricai, Oana Suciu, Delia Ioana Horhat, Călin Marius Popoiu, Ovidiu Adam, Eugen Boia

**Affiliations:** 1 Department of Rehabilitation, Physical Medicine and Rheumatology, “Victor Babes” University of Medicine and Pharmacy Timișoara, 300042 Timișoara, Romania; catan.liliana@umft.ro (L.C.); amaricai.elena@umft.ro (E.A.); oanasuciu78@umft.ro (O.S.); 2 “Louis Turcanu” Emergency Children’s Hospital, 300011 Timișoara, Romania; mcpopoiu@umft.ro (C.M.P.); adamovidiu29@yahoo.com (O.A.); boia.eugen@umft.ro (E.B.); 3 Department of Radiology, “Victor Babes” University of Medicine and Pharmacy Timișoara, 300042 Timișoara, Romania; 4 “Pius Brinzeu” Emergency County Hospital Timisoara, Timisoara 300723, Romania; 5 Department of ENT, “Victor Babes” University of Medicine and Pharmacy Timișoara, 300042 Timișoara, Romania; deliahorhat@yahoo.com; 6 Department of Paediatric Surgery, “Victor Babes” University of Medicine and Pharmacy Timișoara, 300042 Timișoara, Romania

**Keywords:** scoliosis, plantar pressure, stabilometric analysis, vitamin D

## Abstract

(1) Background: Adolescent idiopathic scoliosis (AIS) can be associated with vitamin D deficiency and osteopenia. Plantar pressure and stabilometry offer important information about posture. The objectives of our study were to compare static plantar pressure and stabilometric parameters, serum 25-OH-vitamin D3 and calcium levels, and bone mineral densitometry expressed as z-score in patients with moderate AIS and healthy subjects. (2) Methods: 32 female adolescents (idiopathic S shaped moderate scoliosis, main lumbar curve) and 32 gender and age-matched controls performed: static plantar pressure, stabilometry, serum 25-OH-vitamin D3 and calcium levels, and dual X-ray absorptiometry scans of the spine. (3) Results: In scoliosis patients, significant differences were recorded between right and left foot for total foot, first and fifth metatarsal, and heel loadings. Stabilometry showed a poorer postural control when compared to healthy subjects (*p* < 0.001). Patients had significantly lower vitamin D, calcium levels, and z-scores. Lumbar Cobb angle was significantly correlated with the z-score (r = −0.39, *p* = 0.02), with right foot fifth metatarsal load (r = −0.35, *p* = 0.04), center of pressure CoP_x_ (r = −0.42, *p* = 0.01), CoP displacement (r = 0.35, *p* = 0.04) and 90% confidence ellipse area (r = −0.38, *p* = 0.03). (4) Conclusions: In our study including female adolescents with idiopathic S shaped moderate scoliosis, plantar pressure and stabilometric parameters were influenced by the main scoliotic curve.

## 1. Introduction

Scoliosis is a three-dimensional spinal abnormality. The idiopathic one is the most prevalent type in adolescents (80–90%) [1]. For that matter, adolescent idiopathic scoliosis (AIS) affects between 0.47% and 11.1% of the world population [2]. 

Numerous factors are thought to be involved in the etiology of scoliosis. There seems to be a connection between the appearance of scoliosis and child growth and development, hormone release, posture, and physical activity [3,4,5,6,7,8,9]. The study of Silferi et al. [10] on a fifteen AIS group showed an impairment of the central nervous system. The most affected mechanisms are responsible to manage the integration of visual, proprioceptive, and vestibular data in order to regulate the vertical position and thus the postural stability [10].

Vitamin D is a major factor involved in the bone growth; if deficient during first years of life, it is associated with poor skeletal mineralization, leading to a wide variety of skeletal deformities. While some studies have reported that vitamin D deficiency is frequent in patients with AIS, the modality by which vitamin D influences scoliosis is not established [11].

There are studies that also revealed an important association between scoliosis and osteopenia [12,13,14,15,16,17,18,19]. Other authors reported osteopenia as a cause of scoliosis worsening as it represents a risk of curve progression [20,21]. Moreover, scientific reports showed an increased prevalence of low bone mass in girls diagnosed with AIS [22,23].

Plantar pressure measurement provides useful objective information for physicians; it is an important tool to determine the treatment option for an enhanced clinical outcome [24]. Various studies indicated that the trajectory of center of pressure has a relevant significance for human biomechanics [25,26,27,28]. 

There are only few studies that emphasized the relationship between modified static plantar pressure parameters and scoliosis; most investigations highlighted the importance of center of pressure trajectory in gait [29,30,31,32,33]. 

The hypothesis of our study was the fact that scoliosis can influence the static plantar pressure and stabilometric parameters and may be related with vitamin D deficiency and low bone mineral density.

The main aim of the current research was to compare the static plantar pressure and stabilometric parameters between female adolescents with idiopathic scoliosis and healthy subjects. Another objective was to analyze the serum levels of 25-OH-vitamin D3 and calcium, and bone mineral density in the two groups. To our knowledge there is no study that has assessed the static plantar pressure and stabilometric parameters, bone mass index, and vitamin D altogether in adolescent idiopathic scoliosis.

## 2. Materials and Methods 

### 2.1. Ethics Statement

The current research was achieved in accordance with the Declaration of Helsinki, being approved by Institutional Ethics Committee (“Louis Țurcanu” Emergency Children’s Hospital No 56/05 October 2018). Participation in the study was voluntary. All the participants’ parents gave their written informed consent.

### 2.2. Participants

The parents of 36 children were requested to take part in the study. The children were patients of the Pediatric Surgery Department, “Louis Turcanu” Emergency Children’s Hospital Timișoara, România. The general practitioners mainly addressed the children to the hospital. However, there were some cases in which the parents came to the hospital as they had noticed the poor posture of their children.

Inclusion criteria were: post-menarcheal adolescent girls; idiopathic S shaped scoliosis (right convex thoracic and left convex lumbar curves, main lumbar), with a lumbar Cobb angle between 25–45°. Exclusion criteria were: congenital or other musculoskeletal disorders, neurologic, endocrine, infectious, malignant, and psychiatric diseases. Four patients met the exclusion criteria: clubfoot deformity (2); femur bone cyst (1); hypoparathyroidism (1). 

We enrolled 32 patients. Thirty-two gender and age-matched healthy controls joined the study after finding out information from posters placed at city schools. The controls were healthy adolescents; they did not suffer from any musculoskeletal disorder. Before being considered controls, they needed to have undergone clinical examination. The medical history was also recorded. The students’ parents were asked if they were willing to take part in the study. The permission to place information posters was obtained from the schools.

A sample size analysis was performed using the G*Power 3.1.9.2 software (Christian-Albrechts-Universität Kiel, Kiel, Germany). For a power of 80% beta = 0.2, alpha = 0.05 and an estimated effect size of 0.7, the number of subjects for each group should be at least 26 [34].

The study was conducted between January 14, 2019 and September 30, 2019.

### 2.3. Assessment

Patient and control features were collected. The participants were assessed at “Louis Turcanu” Emergency Children’s Hospital Timișoara, România. The patients were assessed clinically by the same investigator. The protocol of our research implied one assessment for the study group and controls. The following determinations were performed: static plantar pressure analysis and stabilometric analysis, X-rays of the thoracic and lumbar spine (standing position) with the measurement of Cobb angle by a single experienced investigator, serum 25-hydroxyvitamin D level, and bone mineral density measurement. The 2016 SOSORT (Scientific Society on Scoliosis Orthopedic and Rehabilitation Treatment) guidelines define mild scoliosis by a Cobb angle less than 20°; moderate scoliosis is characterized by a Cobb angle between 21° and 35° [35]. All study patients were first time patients diagnosed with scoliosis.

#### 2.3.1. Plantar Pressure and Stabilometric Analysis

For the static plantar pressure assessment, we used a capacitive system (PoData, Chinesport, Italy). It records data of weight distribution, barycenter, and stabilometry [36,37]. The subjects had to stand on the platform, shoeless, with their eyes opened for 20 s. They were advised to look ahead without talking or moving. The testing was considered invalid and repeated if the subject changed the initial position, moved a part of the body (arm/arms, foot, heel, or head), or talked. 

The device registered the plantar pressure distribution on first and fifth metatarsal heads, and calcaneus [36,38]. For each foot, the percentage of body weight was computed. An optimal load of a model subject is distributed as follows: 16.67% of total weight on fifth metatarsal head, 33.33% of total weight on first metatarsal head, and 50% of total weight on heel [36,38]. The measured body center of pressure (CoP) was then examined in contrast with the theoretical one [39,40].

Body center of pressure deviation from hypothetical framework was measured on anterior–posterior (CoP_Y_) and latero–lateral (CoP_X_) axes [36,39,41]. An anterior deviation on the CoP_Y_ axis and a right deviation on the CoP_X_ axis signify a positive value. The system also determined the absolute mean CoP displacement from the ideal position [42]. We analyzed in our study other CoP parameters, namely the CoP path length, the 90% confidence ellipse area, and the maximum CoP speed, as recommended by Nagymate and Kiss [43]. The CoP path length represents the length (measured in millimeters) of the subject’s center of gravity shift during the test. Confidence ellipse area is the area (in mm^2^) of the ellipse containing all the center of gravity points measured and transferred on a system of Cartesian axes; the confidence level is 90%. Maximum CoP speed (expressed in millimeters per second) means the average center of gravity shifting maximum speed.

#### 2.3.2. Laboratory Tests

The blood samples were collected by the same investigator. Venous blood was sampled for determining serum calcium and serum 25-OH-vitamin D3 (25OHD) in the morning, after an 8-hour fasting. Serum calcium was measured by automated biochemistry analyzer Cobasc-501 (Roche Diagnostics, Manheim, Germany). The normal values for serum total calcium were between 8.8 and 10.8 mg/dL (in 2–12-year old children) and between 8.4 and 10.5mg/dL (in 12–18-year old children). The serum 25OHD was determined using the chemiluminescence method (Diasorin, Stillwater, MN, USA). A serum 25OHD level less than 20 ng/mL was defined as Vitamin D deficiency. Vitamin D deficiency was considered severe (25OHD < 5 ng/mL), moderate (25OHD < 10 ng/mL) or mild (25OHD < 20 ng/mL) [44].

#### 2.3.3. Bone Mineral Density Measurement

Dual X-ray absorptiometry (DXA) is frequently used to define osteoporosis [45]. All DXA scans were performed with a Hologic Delphi W (Hologic, Florida, FL, USA) at a single institution. Information from the DXA scans was obtained of the spine, including bone mineral density (g/cm^2^), expressed as z-scores. According to the Official Pediatric Positions of the International Society for Clinical Densitometry, low bone mineral mass or bone mineral density is the favored nomenclature for pediatric DXA reports when BMC or areal BMD z-scores are equal or below −2.0 [46,47]. The DXA scans were performed by a single investigator.

### 2.4. Statistical Analysis

The Shapiro-Wilk test was used for testing data‘s normality. The homogeneity of variances between groups was tested with Levene’s test. Demographic data (age, height, weight, and body mass index) between patients and controls were compared with t-tests and Fisher exact tests. Mann–Whitney tests and t-tests for independent measures were performed to assess the differences between patients and control groups, for non-normal distributed data (vitamin D, Calcium, z-scores, Cobb angles, plantar pressure loads, CoP displacements) and normal distributed data, respectively (CoP path length, 90% confidence ellipse area, and maximum CoP speed). The Wilcoxon test was used to investigate the differences between limbs for the plantar pressure load distribution. The relationship between levels of calcium, vitamin D, Cobb’s angle, and stabilometric parameters was analyzed with the Spearman‘s rank correlation coefficient. The significance level was set at *p* < 0.05 for all tests. The statistical analysis was performed with the MedCalc Statistical Software version 19.1 (MedCalc Software bv, Ostend, Belgium).

## 3. Results

The two groups were homogenous in terms of anthropometrical characteristics (Levene’s test, *p* > 0.05) (Table 1).

The serum vitamin D and calcium levels, DXA z-scores, and Cobb’s angle values are presented in Table 2. Vitamin D and calcium levels were significantly higher in control group, as well as the z-scores.

The results of plantar pressure analysis are presented in Table 3. In the patient group we found significant differences between right and left foot for all the assessed parameters (total foot load, first and fifth metatarsal load, and heel load). In contrast, healthy controls had no differences between right and left foot measurements.

When analyzing the total foot load between children with scoliosis and controls, we found a significantly higher load on the left foot and a significantly lower load on the right foot in the patient group (*p* < 0.0001). The load on the first metatarsal was significantly decreased, while on the fifth metatarsal it was significantly increased in patients in comparison to the controls for both right and left feet. The adolescents with scoliosis had a significantly lower heel load on the right foot. There were no significant differences in left heel loadings between patients and controls (*p* = 0.38).

The stabilometric data are presented in Table 4. In the patient group the barycenter showed a deviation to the left (median −7 mm (−10 to −6)) on the latero–lateral axis and anteriorly (median 4.5 mm (−2 to 10.5)) on the anterior–posterior axis. The deviations were recorded taking into account the ideal barycenter for each subject. There were significant differences between the two groups for all the analyzed parameters, with higher values in the patient group.

The lumbar Cobb angle (the main scoliotic curve) was significantly correlated with the following: the z-score (r = −0.39, *p* = 0.02), the right foot MT5 load (r = −0.35, *p* = 0.04), COP_X_ (r = −0.42, *p* = 0.01), COP displacement (r = 0.35, *p* = 0.04) and 90% confidence ellipse area (r = −0.38, *p* = 0.03).

## 4. Discussion

Our research was focused on the evaluation of the static plantar pressure and stabilometry in female adolescents diagnosed with idiopathic S shaped moderate scoliosis (right convex thoracic and left convex lumbar curves, main lumbar). Our patients had an increased total foot load on the left limb in comparison to the right one (body weight distribution: left foot 54% vs. right foot 46%, *p* < 0.0001). This overload on the left foot corresponds to the left convex lumbar curve. Regarding the comparison of the three sites of weight distribution between the right and the left foot we noticed a higher load on the first and fifth metatarsal heads on the right side, with an increased load on the left calcaneus. When compared to gender and age-matched healthy adolescents, important differences in weight loadings were reported for both limbs. These differences involved the total foot load and the three weight distribution sites, except for the left heel load.

Szulc et al. in their study (2008) carried out on thirty 10-year old girls diagnosed with right-side idiopathic thoracic mild degree scoliosis noticed that a higher pressure was imposed on the hallux and convex front part of the foot [48]. The study by Lee and Shim (2015) compared the dynamic plantar pressure in ten children with scoliosis (initial average Cobb angle of 14.5 ± 1.5°) before and after stabilization exercises. The testing consisted of analyzing eight plantar areas including only the left foot [31]. Our study including 32 patients assessed the static plantar pressure of both the right and left foot, as well as the three main sites of weight distribution. The recorded data were also compared to healthy female adolescents.

In the present research we also studied the deviation of the barycenter on the anterior–posterior and latero–lateral axes. Adolescents diagnosed with S shaped scoliosis with the main lumbar curve had a higher deviation anteriorly and to the left. This finding suggests an elevated support on the left forefoot; the leg corresponds to the lumbar convex curve. The CoP displacement represents a measurement of the body’s neuromuscular need to preserve its vertical stability. Previous studies indicated that the trajectory of CoP is an important biomechanical tool for assessing the postural stability [28,49,50]. Lee et al. (2014) in a study on ten AIS patients found an increased CoP displacement in the scoliosis group compared to the healthy controls [29]. In our research we found significant differences in CoP displacement on a larger group of AIS patients when matched to healthy adolescents. There is also a significant correlation between Cobb angle and CoP displacement. The comparison of stabilometric data between patients and controls shows that scoliosis curve has an important influence on postural stability. There is a significant inverse correlation between lumbar left curve and CoPx lateral deviation. Gauchard et al. recorded that the site of the major curve influences the lateral instability; patients with high major curves had a better postural stability than those with low major curves [51].

In the current study we also compared the serum vitamin D and calcium levels, as well as the bone mineral density between female adolescents with scoliosis and healthy controls. Some studies noticed a possible association between vitamin D levels and scoliosis [2,52]. Balioglu et al. found lower vitamin D levels and a positive correlation between vitamin D levels and calcium levels [53]. In our research all patients had a vitamin D deficiency, although the calcium level was normal. The z-score was below −2 in 46.87% of our patients. 

We found that the lumbar Cobb angle was significantly correlated with the z-score; a higher lumbar scoliosis angle correlates with a lower DXA score. The results are related to Warren et al. (2005) [54]; they concluded that in children with AIS, during the peripubertal period, curve severity and bone mineral density were inversely correlated. They noticed that 13 years and older patients, with moderate or severe scoliosis, had a lower bone mineral density than the healthy controls [54]. In our study, the patients with low bone mineral density were between 13 and 17 years old; 11 and 12 year old adolescents had a normal bone mineral density. The overall patients’ assessment is an important issue in today’s medicine, taking into account laboratory and imaging tests as well as posture related research [55,56,57].

The adolescents included in our study received the instruction for wearing thoracolumbosacral orthosis for 23 h per day. They also started the rehabilitation program and were directed to the pediatric department for the treatment of vitamin D deficiency. Previous researches have reported beneficial effects of conservative treatment in AIS and other musculoskeletal diseases, with long-term consequences on the quality of life [58,59,60,61].

The plantar pressure analysis should contribute to a patient tailored exercise program. This recommendation will have implications for both research and clinical practice. The corrections of the posture and body alignment, as well as the plantar pressure equalization under visual control, were the main targets of the rehabilitation indicated for our patients. The implication for research can be represented by the prognostic value of the plantar pressure in the evolution of scoliosis.

The current research has some limitations. We included a relatively small number of female adolescents with moderate scoliosis, with main left convex lumbar curve. Additional studies are required to analyze the effects of all types of scoliosis on plantar pressure in a balanced gender distribution.

## 5. Conclusions

In our study including female adolescents diagnosed with idiopathic S shaped moderate scoliosis, the static plantar pressure and stabilometric parameters were influenced by the main scoliotic curve. Important differences in weight distribution and stability were recorded in comparison to healthy controls. These data, associated with the vitamin D deficiency and the high percentage of low bone mineral density, may contribute to the follow-up of body alignment of patients with scoliosis undergoing conservative treatment.

## Figures and Tables

**Table 1:** Demographic characteristics.

Parameters	Patients (*n* = 32)	Controls (*n* = 32)
Age (years) ^1^	14.75 (1.34)	14.75 (1.34)
Height (cm) ^1^	159.63 (10.15)	162.91 (8.51)
Weight (kg) ^1^	47.03 (10.32)	51.47 (7.78)
BMI (kg/m2) ^1^	18.3 (2.99)	19.26 (1.3)
Environment		
Rural, *n* (%)	14 (43.75)	15 (46,87)
Urban, *n* (%)	18 (56.25)	17 (53.12)

^1^ Data are presented as mean (SD). BMI—body mass index

**Table 2:** Biochemical, mineral density, and scoliosis parameters.

Parameters	Patients	Controls	*p*
Vitamin D (ng/mL)	17.7 (14.92–20.03)	33.95 (29.95–41.87)	<0.0001
Calcium (mg/dL)	2.48 (2.38–2.59)	2.67 (2.59–2.71)	<0.0001
DXA z-score	−1.8 (−2.3 to −0.85)	1 (0.4–1.75)	<0.0001
Lumbar Cobb angle (°)	31 (27–38)	-	NA
Dorsal Cobb angle (°)	20 (15.5–27)	-	NA

Data are presented as median and interquartile range. NA–notapplicable

**Table 3:** Plantar pressure load distribution in patients and controls.

Parameters	Patients (*n* = 32)	Controls (*n* = 32)	*p* *
Right foot load (%)	46 (36–47)	51 (49–51)	<0.0001
Right foot MT1 load (%)	31 (23.5–34)	36 (35–37.5)	<0.0001
Right foot MT5 load (%)	27.5 (26–34)	15 (11.5–16)	<0.0001
Right foot, heel load (%)	40.5 (38–42.5)	50 (49–51)	<0.0001
Left foot load (%)	54 (53–64)	49 (49–51)	<0.0001
Left foot, MT1 load (%)	23.5 (17–25)	36 (34–37)	<0.0001
Left foot, MT5 load (%)	25 (21–35)	14 (12–15)	<0.0001
Left foot, heel load (%)	53 (47–55)	51 (49.5–51)	0.38

MT1: first metatarsal, MT5: fifth metatarsal; all data are presented as median and interquartile range; *p* * relates to differences between patients and controls

**Table 4:** Plantar pressure load distribution in patients and controls.

ParametersTitle	Patients (*n* = 32)	Controls (*n* = 32)	*p*
CoP_X_ *	−7 (−10 to −6)	0.5 (0–1.5)	<0.0001
CoP_Y_ *	4.5 (−2 to 10.5)	0.5 (−0.75 to 0.5)	0.001
CoP displacement(mm) *	12.53 (7.44–18.44)	1.5 (0.85–2)	<0.001
CoP path length (mm) **	345 (53.80)	208.31 (28.67)	<0.0001
90% confidence ellipse area (mm^2^) **	91.18 (29.29)	56.93 (18.96)	<0.0001
Maximum CoP speed (mm/s) **	88.59 (19.22)	54.71 (14.10)	<0.0001

* Data are presented as median (interquartile range); ** Data are presented as mean (standard deviation); CoP: center of pressure; *p* relates to differences between patients and controls
